# The Importance of General Self-Efficacy for the Quality of Life of Adolescents with Chronic Conditions

**DOI:** 10.1007/s11205-012-0110-0

**Published:** 2012-06-29

**Authors:** Jane M. Cramm, Mathilde M. H. Strating, Marij E. Roebroeck, Anna P. Nieboer

**Affiliations:** 1 Institute of Health Policy and Management, Erasmus University, P.O. Box 1738, 3000 DR Rotterdam, The Netherlands; 2 Department of Rehabilitation Medicine and Physical Therapy, Erasmus University Medical Centre, Rotterdam, The Netherlands

**Keywords:** Self-efficacy, Quality of Life, Chronic condition, Adolescent, Parent

## Abstract

We investigated the influence of general self-efficacy perceived by adolescents with chronic conditions and parents on quality of life. This cross-sectional study used the general self-efficacy scale and DISABKIDS condition-generic module to survey adolescents (92/293; 31 %) with type I diabetes, juvenile rheumatoid arthritis, cystic fibrosis, kidney/urological conditions, and neuromuscular disorders; and parents (121/293; 41 %). Self perceived and parents’ perceived general self-efficacy of adolescents was compared using paired t-tests, and adolescents’ quality of life and general self-efficacy were compared among conditions using analysis of variance. Bivariate correlations between general self-efficacy and quality of life were identified, and multiple regression sought predictors of quality of life after controlling for background variables. Social quality of life was lowest among those with neuromuscular disorders. General self-efficacy was highest among adolescents with cystic fibrosis and lowest among those with urological conditions. Parents’ perceptions of general self-efficacy were higher than adolescents’ (*p* ≤ 0.05), although absolute differences were small. General self-efficacy perceived by parents and adolescents was related to emotional, physical, and social quality of life. Adolescents’ perceived self-efficacy predicted all quality of life domains. Parents’ perceptions of the adolescents self-efficacy predicted the adolescents’ social quality of life (β = 0.19; *p* ≤ 0.01). General self-efficacy of adolescents with chronic conditions as perceived by themselves and their parents is important for adolescents’ quality of life. Interventions to improve general self-efficacy should benefit quality of life among these adolescents.

## Introduction

Self-efficacy is the belief in one’s competence to attempt difficult or novel tasks, and to cope with adversity arising from specific demanding situations (Cross et al. 2006; Luszczynska et al. 2005; Scholz et al. 2002). People with high self-efficacy choose to perform more challenging tasks; they set themselves higher goals and stick to them. Actions are pre-shaped in thought, and once an action has been taken, highly self-efficacious people invest more effort and persist longer than do those with low self-efficacy (Bandura 1997, 1999). When setbacks occur, they recover more quickly and remain committed to their goals. Thus, self-efficacy is an important factor in coping with the challenges and demands presented by a chronic condition.

Everyday healthcare and studies investigating adolescents with chronic conditions typically apply a disease-specific approach. Studies including adolescents with different conditions or cross-referencing between studies of different conditions are uncommon (Sawyer et al. 2007). However, a wide range of chronic conditions encompasses many comparable tasks, such as managing symptoms and treatment, forming relationships with care providers, maintaining a positive self-image, relating to family and friends, and preparing for an uncertain future (Moos and Holahan 2007). Appreciation of the similarities and differences among specific diseases could increase our understanding of how diseases can differentially affect adolescents (Sawyer et al. 2007). Generic measures facilitate comparison among adolescents with different conditions; therefore, general self-efficacy may be useful in predicting health behaviors and quality of life across conditions.

When faced with a chronic disease, adolescents are expected to take actions (e.g., taking medication, engaging in daily physical activity) to reduce the effects of the disease on their physical quality of life; self-efficacy may play an important role in such actions. Research has shown that general self-efficacy predicts adherence (taking medication), health behavior (physical activities), effective pain management, and disease management (Andre et al. 1999; Bar-Mor et al. 2000; Es et al. 2002; Griva et al. 2000; Luszczynska et al. 2004, 2005a, b, 2010; Morisky et al. 2001; Ott et al. 2000; Schwarzer and Fuchs 1996). General self-efficacy may also be important for emotional quality of life, since it is clearly related to positive and negative emotions. Highly self-efficacious adolescents with chronic conditions may be better able to cope with stressful situations arising from their conditions (Griva et al. 2000; Jerusalem and Schwarzer 1992), which may reduce feelings of stress and enhance positive emotions (Bandura 1997). Individuals with perceived self-inefficacy suffer distress and negative emotions, such as anxiety and depression (Bandura 1997; Schwarzer 1992). Furthermore, general self-efficacy may be important for social quality of life. Chronic diseases in adolescence can represent a major psychosocial burden. Social disruption, social stigma and marginalization, and changes in plans and expectations about the future can be substantial challenges to emotional as well as social quality of life (DiNapoli and Murphy 2002; Kyngas 2004). Berntsson et al. (2005) showed that acceptance of one’s chronic disease and feelings of support from family, friends, healthcare professionals, and society are crucial for the quality of life of adolescents with chronic conditions. A belief in one’s high level of efficacy is related to the expansion of satisfying social relations (Bandura 1997), which may be beneficial to quality of life outcomes. In physically disabled young people, self-efficacy was found to be an important predictor of social participation, in addition to lower levels of disability, fatigue, and pain (Bent et al. 2001). In a sample of adults with bilateral cerebral palsy, a higher level of general self-efficacy was correlated with better social participation and higher quality of life (van der Slot et al. 2010).

Considering the importance of general self-efficacy on quality of life outcomes among adolescents with different chronic conditions, and the lack of research investigating general self-efficacy and quality of life among adolescents across different chronic conditions, this study aimed to investigate the influence of general self-efficacy on physical, emotional, and social quality of life outcomes, as perceived by adolescents with a variety of chronic conditions.

Parents’ perceptions of self-efficacy in adolescents with chronic conditions may also affect adolescents’ quality of life outcomes. For example, parents’ behavior has been found to be positively related to adolescents’ general self-efficacy and effective disease management (Ott et al. 2000), which may positively affect quality of life. Another study found that parents’ attitudes were related to general self-efficacy, and thus increased physical activity, in adolescents with trivial, mild, or moderate congenital cardiac malformations (Bar-Mor et al. 2000), which may also lead to higher quality of life. Based on these findings and given the importance of social context for self-efficacy (Bandura 1997), we expected to find a relationship between parents’ perceptions of their children’s self-efficacy and the children’s quality of life. Therefore, we additionally investigated whether parents’ perceptions of self-efficacy in their adolescent children correlated with their children’s perceptions of self-efficacy and quality of life outcomes.

## Methods

### Setting and Design

This study surveyed adolescents with type I diabetes (*n* = 229); juvenile rheumatoid arthritis (*n* = 132); cystic fibrosis (*n* = 24); kidney conditions, such as renal insufficiency/kidney failure or kidney transplantation (*n* = 15); urological conditions, such as imperforate anus with malfunctions in the anus, rectum, urethra, and bladder, or exstrophy of the bladder (a congenital anomaly in which part of the urinary bladder is present outside the body) (*n* = 22); and neuromuscular disorders (*n* = 38); the latter used home ventilation. A total of 92/293 (31 % response rate) adolescents with chronic conditions and 121/293 (41 % response rate) parents completed the questionnaire. Eligible participants were 12–25-year-olds in active pediatric follow-up (at least one contact moment in the past year) of participating hospital departments. Adolescents who had been transferred to adult care or had a documented diagnosis of intellectual impairment were excluded from this study. Approval for the study was obtained from the Erasmus Medical Centre Institutional Review Board. Eligible adolescents and parents received written information and a unique access code, and were invited to complete an online questionnaire. Non-respondents received a postal reminder after 2 weeks, including a printed copy of the questionnaire.

### Measures

The Dutch version of the 10-item general self-efficacy scale, developed by Schwarzer and Jerusalem (1995), was used to assess adolescents’ and parents’ perceptions of self-efficacy in adolescents with chronic conditions. A typical item is, “I can always manage to solve difficult problems if I try hard enough.” Responses are structured on a four-point scale: 1, not at all true; 2, hardly true; 3, moderately true; and 4, exactly true. A total score (10–40) is obtained by summing the responses to each of the 10 items. Several studies have confirmed the high reliability, stability, and construct validity of the general self-efficacy scale (Jerusalem and Schwarzer 1992; Leganger et al. 2000; Luszczynska et al. 2005a, b; Schwarzer 1992; Schwarzer and Born 1997; Schwarzer et al. 1997a, b, 1999; Sherer et al. 1982). Cronbach’s alpha values of the general self-efficacy scale among adolescents (0.87) and parents (0.94) in this study both indicated internal reliability.

Quality of life was assessed with the DISABKIDS condition-generic module questionnaire (Petersen et al. 2005), which consists of 37 items grouped into six dimensions: ‘independence’, ‘physical limitation’, ‘social inclusion’, ‘social exclusion’, ‘emotion’, and ‘medication’. The six dimensions are conceptually associated with three higher-order domains: *emotional*, *physical*, and *social* quality of life. The *emotional* domain incorporates the positive aspect of independent living (‘independence’ dimension), as well as all kinds of emotional reactions to having a chronic condition (‘emotional’ dimension). The *physical* domain includes physical symptoms and limitations due to the chronic condition (‘physical limitation’ dimension), as well as the impact of taking medicine (in the form of medication, injections, etc.; ‘medication’ dimension). The *social* domain includes aspects of stigma (‘social exclusion’ dimension) and support from friends, family, and others (‘social inclusion’ dimension) (Schmidt et al. 2006). Responses to each item are structured using a five-point Likert scale: 1, never; 2, seldom; 3, quite often; 4, very often; and 5, always. Domain scores were transformed linearly to a 0–100 scale using a transformation formula: (actual raw score − lowest possible raw score)/possible raw score. This transformation converts the lowest and highest possible scores to zero and 100 respectively. Scores between these values represent the percentage of the total possible score achieved, with 100 indicating the highest quality of life. Cronbach’s alpha values of the social (0.79), emotional (0.90), and physical (0.84) domains of the DISABKIDS condition-generic module in this study population all indicated internal reliability.

The questionnaire further asked respondents to provide information about background variables, such as age, gender, educational level, type of chronic condition, and level of physical functioning. To account for the severity of chronic conditions, we used the SF-20 physical functioning scale (Carver et al. 1999; Kempen 1992). The scale was transformed to range from 0 to 100, with higher scores indicating higher levels of physical functioning. Educational level was assessed on an 11-point scale, ranging from primary education (0) to university (11). We dichotomized education into high (higher general continued education, preparatory scientific education, higher professional education, university = 1) and other (0).

### Statistical Analyses

We investigated the relationship between self-efficacy as perceived by adolescents with chronic conditions and their parents on the emotional, physical, and social domain scores of the DISABKIDS condition-generic module instrument. Descriptive analyses included the calculation of means and standard deviations. Differences in the general self-efficacy scores provided by adolescents with chronic conditions and parents were established with two-tailed paired *t* tests, and quality of life and general self-efficacy scores were compared among adolescents with different chronic conditions using analysis of variance. Bivariate correlations between general self-efficacy scores and quality of life domains were first identified. Multiple regression analyses were then performed to reveal significant predictors of quality of life domains after controlling for age, gender, education, physical functioning, and type of chronic condition. All statistical analyses were conducted with SPSS software (ver. 17.0; SPSS, Inc., Chicago, IL, USA).

## Results

About half (53 %) of the adolescents were female and their mean age was 17 ± 2.2 (range 12–24) years (Table 1). Sixteen percent of the adolescents with chronic conditions were highly educated. Half (50 %) of the adolescents reported having diabetes, 29 % had juvenile rheumatoid arthritis, 8 % had neuromuscular disorders, 5 % had cystic fibrosis, 5 % had urological conditions, and 3 % had kidney conditions. The majority (86 %) of participating parents were mothers, and 14 % were fathers. Parents’ mean age was 49 ± 5.1 (range 37–67) years.

**Table 1:** Descriptives of background characteristics and general self-efficacy

	Percentage or mean ± SD
*Background characteristics*	
Age	17 ± 2.2
Gender (female)	53 %
Education (high)	16 %
Physical functioning	77.0 ± 30.2
*General self*-*efficacy scores*	
General self-efficacy perceived by adolescents	30.3 ± 6.1
General self-efficacy perceived by parents	29.3 ± 4.7

*SD* standard deviation

Table 2 shows differences among chronic conditions in quality of life domain scores and general self-efficacy as perceived by adolescents with chronic conditions and their parents. Social domain scores varied among chronic conditions (F_group_ = 5.668; *p* ≤ 0.01). Although adolescents with diabetes and cystic fibrosis reported the highest social quality of life outcomes, these values did not differ significantly from the mean overall social quality of life score. Those with neuromuscular disorders using home ventilation reported the lowest social quality of life, which was significantly lower than the overall mean social quality of life score (*p* ≤ 0.001). Two-tailed *t*-tests showed that parents’ perceptions of adolescents’ general self-efficacy were significantly higher than adolescents’ perceived self-efficacy (30.3 ± 6.1 vs. 29.3 ± 4.7; *p* ≤ 0.05), although absolute differences were small. General self-efficacy as perceived by adolescents with chronic conditions (F_group_ = 2.732; *p* ≤ 0.05) and their parents (F_group_ = 3.690; *p* ≤ 0.01) varied among chronic conditions; general self-efficacy scores were highest among adolescents with cystic fibrosis (significantly higher than the mean overall score; *p* ≤ 0.001), and lowest among those with urological conditions (significantly lower than the mean overall score; *p* ≤ 0.01). Level of physical functioning also varied among chronic conditions (F_group_ = 16.030; *p* ≤ 0.001). Adolescents with neuromuscular disorders reported the lowest levels of physical functioning, followed by adolescents with urological conditions. Adolescents with cystic fibrosis and diabetes reported the highest levels of physical functioning.

**Table 2:** Differences in quality of life domain scores (DISABKIDS condition-generic module questionnaire) and general self-efficacy scores (General-Self-Efficacy scale) among adolescents with different chronic conditions

	JRA	NMDs	Diabetes	Cystic fibrosis	Urological conditions	Kidney conditions	Total	F	*p*
*Emotional quality of life*									
*n*	54	21	112	12	18	9	226	1.327	0.254
Mean	81.4	73.4	76.3	79.4	73.2	75.6	77.1		
SD	15.1	14.8	17.0	11.0	15.4	15.5	16.0		
*Physical quality of life*									
*n*	38	16	110	14	13	9	200	0.858	0.511
Mean	64.8	65.9	67.3	74.6	71.0	71.8	67.7		
SD	17.4	17.2	18.7	16.9	9.6	15.7	17.6		
*Social quality of life*									
*n*	54	21	113	14	18	9	229	5.668	≤0.001
Mean	79.8	66.6	81.0	83.4	71.5	76.2	78.6		
SD	15.4	12.2	11.9	9.1	17.9	15.9	14.0		
*General self-efficacy perceived by adolescents*									
*n*	54	21	113	14	18	9	229	2.732	0.020
Mean	29.9	29.4	29.8	32.8	26.9	28.7	29.7		
SD	4.6	7.6	3.9	2.8	3.8	6.2	4.7		
*General self-efficacy perceived by parents*									
*n*	70	21	139	22	18	11	281	3.690	0.003
Mean	30.2	27.6	30.7	32.1	25.7	26.4	29.9		
SD	6.5	8.8	5.8	5.3	7.9	29.9	6.6		
*Physical functioning*									
*n*	51	5	111	14	18	9	208	16.030	≤0.001
Mean	69.0	23.3	87.4	90.5	42.6	72.2	77.0		
SD	30.0	34.6	21.6	14.2	39.7	25.0	30.2		

*JRA* juvenile rheumatoid arthritis, *NMDs* neuromuscular disorders, *SD* standard deviation

Univariate analyses showed that adolescents’ perceived general self-efficacy was significantly related to emotional, physical, and social quality of life (all at *p* ≤ 0.001; Table 3). Adolescent’s general self-efficacy as perceived by parents was also significantly related to their children’s emotional (*p* ≤ 0.01), physical (*p* ≤ 0.05), and social (*p* ≤ 0.001) quality of life. Higher levels of physical functioning were significantly related to emotional, physical, and social quality of life (all at *p* ≤ 0.001). Higher educational level was also significantly related to emotional (*p* ≤ 0.05), physical (*p* ≤ 0.05), and social (*p* ≤ 0.01) quality of life. No significant relationships were found between quality of life domain scores and age or gender.

**Table 3:** Associations among background characteristics, general self-efficacy, and emotional, physical and social quality of life in adolescents with chronic conditions

	Emotional quality of life	Physical quality of life	Social quality of life
*Background characteristics*			
Age	−0.07	0.02	0.03
Gender (female)	−0.12	−0.09	−0.06
Education (high)	0.14*	0.15*	0.18**
Physical functioning	0.36***	0.28***	0.55***
*General self*-*efficacy scores*			
General self-efficacy perceived by adolescents	0.41***	0.33***	0.39***
General self-efficacy perceived by parents	0.18**	0.15*	0.38***

*SD* standard deviation. *** *p* ≤ 0.001; ** *p* ≤ 0.01; * *p* ≤ 0.05 (two-tailed)

Table 4 displays quality of life predictors assessed by multiple regression analyses. After controlling for age, gender, educational level, physical functioning, and type of chronic condition, general self-efficacy as perceived by adolescents with chronic conditions predicted their emotional (β = 0.29; *p* ≤ 0.001) and physical (β = 0.22; *p* ≤ 0.05) quality of life. General self-efficacy as perceived by adolescents with chronic conditions (β = 0.20; *p* ≤ 0.01) and their parents (β = 0.19; *p* ≤ 0.01) predicted adolescents’ social quality of life.

**Table 4:** Quality of life predictors as assessed by multiple regression analyses

	Emotional quality of life	Physical quality of life	Social quality of life
*Background characteristics*			
Age	−0.04	0.06	0.05
Gender (female)	−0.04	−0.00	0.00
Education (high)	0.14	0.14	0.12
Physical functioning	0.34***	0.25**	0.46***
*Chronic conditions*			
JRA	0.18	−0.10	0.15
NMDs	0.09	0.02	0.01
Diabetes	−0.11	−0.17	0.02
Cystic fibrosis	−0.05	−0.05	0.01
Urological conditions	0.11	0.13	0.11
*General self*-*efficacy scores*			
General self-efficacy perceived by adolescents	0.29***	0.22*	0.20**
General self-efficacy perceived by parents	0.07	0.09	0.19**
Adjusted R^2^	22.9 %	9.4 %	36.6 %
F	4.972	2.265	8.859

*JRA* juvenile rheumatoid arthritis, *NMDs* neuromuscular disorders. Kidney conditions was used as the reference group for chronic conditions

*** *p* ≤ 0.001; ** *p* ≤ 0.01; * *p* ≤ 0.05 (two-tailed)

## Discussion

This study evaluated the effect of general self-efficacy as perceived by adolescents with chronic conditions and their parents on the emotional, physical, and social domains of quality of life among adolescents with diabetes, juvenile rheumatoid arthritis, neuromuscular disorder, cystic fibrosis, kidney conditions, and urological conditions. Physical and emotional quality of life did not differ across these chronic conditions. Social quality of life, however, did differ across conditions; adolescents with neuromuscular disorders reported the lowest quality of life. Low levels of physical functioning and the use of home ventilation limits the possibility of participating in social activities for adolescents with neuromuscular disorders (McDonald 2002), which may explain their low social quality of life. Furthermore, this study showed that general self-efficacy was lowest among those with urological conditions, such as imperforate anus. These results are consistent with those of previous studies showing lower self-esteem and confidence among adolescents with such conditions, as many of their physical problems (i.e., odors caused by incontinence) are socially unacceptable. Studies of the long-term outcome of such malformations have found that the majority of patients with these conditions had behavioral problems, and 15 % expressed suicidal thoughts (Hamid et al. 2007; Ludman et al. 1994).

The mean general self-efficacy score (29.3) of adolescents with chronic conditions in our sample was comparable to those of adolescents with physical disabilities (range 28.0–35.0) (Bent et al. 2002) and lower, but not substantially lower, than those found among average students in various countries (range 29.4–33.4) (Luszczynska et al. 2005a, b). Thus, despite their chronic condition, the adolescents in our sample perceived themselves to be capable of dealing with difficult and emergent situations.

Our findings confirmed our expectations that general self-efficacy as perceived by adolescents with chronic conditions may affect their physical, emotional, and social quality of life. We found significant relationships between each of these quality of life domains and general self-efficacy after controlling for age, gender, education, physical functioning, and type of chronic condition. These findings underscore the importance of interventions that aim to improve general self-efficacy among adolescents with chronic conditions, such as those that seek to enhance confidence and the ability to deal effectively with difficult and/or unexpected events.

We found that parents’ perceptions of their children’s general self-efficacy were somewhat higher than adolescents’ perceptions. This may be explained by parents’ more positive perceptions of their children’s self-efficacy in light of their age and chronic condition. The results of multivariate analysis showed that parents’ perceptions of their children’s general self-efficacy affected adolescents’ social quality of life outcomes, but had no significant effect on physical or emotional quality of life. It may be more difficult for parents to influence their children’s physical and emotional quality of life than their social quality of life. Parents who feel that their children are capable of dealing with complex situations may give them more freedom while growing up and allow them to lead more active social lives. Parents could stimulate their children to make their own decisions, for example by giving them more responsibilities. This relationship is probably dynamic; parents of adolescents with chronic conditions with low social quality of life will probably also perceive their children as having lower general self-efficacy.

The limitations of this study should be considered when interpreting the findings. Most importantly, the data collected were cross-sectional; as a result, causal relationships could not be inferred. We only investigated general self-efficacy and quality of life perceived by adolescents with chronic conditions and their parents at one measurement point. While our study showed that general self-efficacy of adolescents with chronic conditions had an important effect on quality of life outcomes, we did not investigate whether interventions aiming to enhance general self-efficacy actually improved quality of life. Furthermore, our model demonstrated high levels of explained variance in the social and emotional quality of life domains, but revealed relatively low explained variance in the physical quality of life domain. Other factors, such as whether or not the condition is life-threatening, progressive, or very unstable, probably affect physical quality of life. Finally, the large majority of participating parents were mothers, preventing us from comparing differences in the perception of children’s general self-efficacy between mothers and fathers or assessing the possible differential effects on their children’s quality of life.

We can conclude that greater general self-efficacy as perceived by adolescents with chronic conditions is significantly related to their physical, social, and emotional quality of life. Furthermore, greater general self-efficacy as perceived by the parents of adolescents with chronic conditions may also improve their social quality of life. These findings are important for professionals and healthcare organizations aiming to improve quality of life in adolescents with chronic conditions. Interventions that aim to improve general self-efficacy are expected to be beneficial.

## References

[CR1] Andre M, Hedengren E, Hagelberg S, Stenstrom CH (1999). Perceived ability to manage juvenile chronic arthritis among adolescents and parents: Development of a questionnaire to assess medical issues, exercise, pain, and social support. Arthritis Care and Research.

[CR2] Bandura A (1997). Self-efficacy: The exercise of control.

[CR3] Bandura A, Pervin L, John O (1999). A social cognitive theory of personality. Handbook of personality.

[CR4] Bar-Mor G, Bar-Tal Y, Krulik T, Zeevi B (2000). Self-efficacy and physical activity in adolescents with trivial, mild or moderate congenital cardiac malformations. Cardiology in the Young.

[CR5] Bent N, Jones A, Molloy I, Chamberlain MA, Tennant A (2001). Factors determining participation in young adults with a physical disability: A pilot study. Clinical Rehabilitation.

[CR6] Bent N, Tennant A, Swift T, Posnett J, Scuffham P, Chamberlain MA (2002). Team approach versus ad hoc health services for young people with physical disabilities: A retrospective cohort study. The Lancet.

[CR7] Berntsson L, Berg M, Brydolf M, Hellström A-L (2005). Adolescents’ experiences of well-being when living with a long-term illness or disability. Scandinavian Journal of Caring Sciences.

[CR8] Carver DJ, Chapman CA, Thomas VS, Stadnyk KJ, Rockwood K (1999). Validity and reliability of the Medical Outcomes Study Short Form-20 questionnaire as a measure of quality of life in elderly people living at home. Age and Ageing.

[CR9] Cross MJ, March LM, Lapsley HM, Byrne E, Brooks PM (2006). Patient self-efficacy and health locus of control: Relationships with health status and arthritis-related expenditure. Rheumatology (Oxford).

[CR10] DiNapoli PP, Murphy D (2002). The marginalization of adolescents with chronic conditions. Nursing Clinics of North America.

[CR11] Es SM, Kaptein AA, Bezemer PD, Nagelkerke AF, Colland VT, Bouter LM (2002). Predicting adherence to prophylactic medication in adolescents with asthma: an application of ASE-model. Patient Education and Counseling.

[CR12] Griva K, Myers LB, Newman S (2000). Illness perceptions and self-efficacy beliefs in adolescents and young adults with insulin dependent diabetes mellitus. Psychology & Health.

[CR13] Hamid CH, Holland AJ, Martin HC (2007). Long-term outcome of anorectal malformations: The patient perspective. Pediatric Surgery International.

[CR14] Jerusalem M, Schwarzer R, Schwarzer R (1992). Self-efficacy as a resource factor in stress appraisal. Self-efficacy: Thought control of action.

[CR15] Kempen GI (1992). Assessment of health status of the elderly. Application of a Dutch version of the MOS scale. Tijdschrift voor Gerontologie en Geriatrie.

[CR16] Kyngas H (2004). Support network of adolescents with chronic disease: Adolescents’ perspective. Nursing & Health Sciences.

[CR17] Leganger A, Kraft P, Røysamb E (2000). Perceived self-efficacy in health behavior research: Conceptualisation, measurement and correlates. Psychology & Health.

[CR18] Ludman L, Spitz L, Kiely EM (1994). Social and emotional impact of faecal incontinence after surgery for anorectal abnormalities. Archives of Disease in Childhood.

[CR19] Luszczynska A, Gibbons FX, Piko B, Tekozel M (2004). Self-regulatory cognitions, social comparison, and perceived peers’ behaviors as predictors of nutrition and physical activity: A comparison among adolescents in Hungary, Poland, Turkey, and USA. Psychology & Health.

[CR20] Luszczynska A, Scholz U, Schwarzer R (2005). The general self-efficacy scale: Multicultural validation studies. The Journal of Psychology.

[CR21] Luszczynska A, Gutiérrez-Doña B, Schwarzer R (2005). General self-efficacy in various domains of human functioning: Evidence from five countries. International Journal of Psychology.

[CR22] Luszczynska A, Cao DS, Mallach N, Pietron K, Mazurkiewicz M, Schwarzer R (2010). Intentions, planning, and self-efficacy predict physical activity in Chinese and Polish adolescents: Two moderated mediation analyses. International Journal of Clinical and Health Psychology.

[CR23] McDonald CM (2002). Physical activity, health impairments, and disability in neuromuscular disease. American Journal of Physical and Medical Rehabilitation.

[CR24] Moos RH, Holahan CJ, Martz E, Livnah H (2007). Adaptive tasks and methods of coping with illness and disability. Coping with chronic disease and disability: Theoretical, empirical, and clinical aspects.

[CR25] Morisky DE, Malotte CK, Ebin V, Davidson P, Cabrera D, Trout PT (2001). Behavioral interventions for the control of tuberculosis among adolescents. Public Health Reports.

[CR26] Ott J, Greening L, Palardy N, Holderby A, DeBell WK (2000). Self-efficacy as a mediator variable for adolescents’ adherence to treatment for insulin-dependent diabetes mellitus. Children’s Health Care.

[CR27] Petersen C, Schmidt S, Power M, Bullinger M, the DISABKIDS Group (2005). Development and pilot-testing of a health-related quality of life chronic generic module for children and adolescents with chronic health conditions: A European perspective. Quality of Life Research.

[CR28] Sawyer SM, Drew S, Yeo MS, Britto MT (2007). Adolescents with a chronic condition: Challenges living, challenges treating. Lancet.

[CR29] Schmidt S, Debensason D, Mühlan H, Petersen C, Power M, Simeoni MC (2006). The DISABKIDS generic quality of life instrument showed cross-cultural validity. Journal of Clinical Epidemiology.

[CR30] Scholz U, Dona BG, Sud S, Schwarzer R (2002). Is general self-efficacy a universal construct? Psychometric findings from 25 countries. European Journal of Psychological Assessment.

[CR31] Schwarzer R (1992). Self-efficacy: Thought control of action.

[CR32] Schwarzer R, Born A (1997). Optimistic self-beliefs: Assessment of general perceived self-efficacy in thirteen cultures. World Psychology.

[CR33] Schwarzer R, Fuchs R, Conner M, Norman P (1996). Self-efficacy and health behaviors. Predicting health behavior: Research and practice with social cognition models.

[CR34] Schwarzer, R., & Jerusalem, M. (1995). Generalized Self-Efficacy scale. In J. Weinmann, S. Wright, & M. Johnston (Eds.), *Measures in health psychology: A user’s portfolio. Causal and control beliefs* (pp. 35–37). Windsor: NFER-NELSON.

[CR35] Schwarzer R, Bässler J, Kwiatek P, Schröder K, Zhang JX (1997). The assessment of optimistic self-beliefs: Comparison of the German, Spanish, and Chinese versions of the General Self-Efficacy scale. Applied Psychology: An International Review.

[CR36] Schwarzer R, Born A, Iwawaki S, Lee Y-M, Saito E, Yue X (1997). The assessment of optimistic self-beliefs: comparison of the Chinese, Indonesian, Japanese and Korean versions of the General Self-Efficacy scale. Psychologia: An International Journal of Psychology in the Orient.

[CR37] Schwarzer R, Mueller J, Greenglass E (1999). Assessment of perceived general self-efficacy on the internet: Data collection in cyberspace. Anxiety, Stress, and Coping.

[CR38] Sherer M, Maddux JE, Mercandante B, Prentice-Dunn S, Jacobs B, Rogers RW (1982). The Self-Efficacy Scale: Construction and validation. Psychological Reports.

[CR39] van der Slot WM, Nieuwenhuijsen C, van den Berg-Emons RJ, Wensink-Boonstra AE, Stam HJ, Roebroeck ME (2010). Participation and health-related quality of life in adults with spastic bilateral cerebral palsy and the role of self-efficacy. Journal of Rehabilitation Medicine.

